# Correspondence between the habitat of the threatened pudú (Cervidae) and the national protected-area system of Chile

**DOI:** 10.1186/s12898-015-0055-7

**Published:** 2016-01-07

**Authors:** Melissa Pavez-Fox, Sergio A. Estay

**Affiliations:** Instituto de Ciencias Ambientales y Evolutivas, Facultad de Ciencias, Universidad Austral de Chile, Valdivia, Chile; Magíster en Ciencias Biológicas mención Neurociencia, Facultad de Ciencias, Universidad de Valparaíso, Valparaíso, 2360102 Chile; Center of Applied Ecology and Sustainability (CAPES), Facultad de Ciencias Biológicas, Pontificia Universidad Católica de Chile, Santiago, 6513677 Chile

**Keywords:** Conservation, Niche modeling, Protected-area networks, Temperate rain forest, Threatened species

## Abstract

**Background:**

Currently, many species are facing serious conservation problems due to habitat loss. The impact of the potential loss of biodiversity associated with habitat loss is difficult to measure. This is particularly the case with inconspicuous species such as the threatened pudú (*Pudu puda*), an endemic Cervidae of temperate forests of Chile and Argentina. To evaluate the effectiveness of the Chilean protected-area system in protecting the habitat of the pudú, we measured the congruence between this specie’s potential distribution and the geographical area occupied by the protected areas in central and southern Chile. The measurements of congruency were made using the Maxent modeling method.

**Results:**

The potential habitat of the pudú was found to be poorly represented in the system (3–8 %) and even the most suitable areas for the species are not currenly protected. According to these results, the protected area network cannot be considered as a key component of the conservation strategy for this species.

**Conclusions:**

The results presented here also serve as a guide for the reevaluation of current pudú conservation strategies, for the design of new field studies to detect the presence of this species in human-disturbed areas or remaining patches of native forest, and for the implementation of corridors to maximize the success of conservation efforts.

**Electronic supplementary material:**

The online version of this article (doi:10.1186/s12898-015-0055-7) contains supplementary material, which is available to authorized users.

## Background

Several emblematic species are currently facing serious conservation problems due to the loss and degradation of their habitat caused by the expansion of the human population [1]. The impact of habitat loss on biodiversity cannot always be estimated. For example, the effect of habitat loss on inconspicuous species, which are difficult to detect or inhabit inaccessible places, cannot be easily measured. Furthermore, the lack of basic information about their life histories of inconspicuous species makes planning for conservation yet more difficult [2].

The pudú, an inconspicuous species endemic to the temperate forests of Chile and Argentina (*Pudu puda*), is one of the smallest deers in the world and one of the least studied mammals of Chilean forest fauna [3]. According to the IUCN [4], the conservation status of the pudú is Vulnerable with an estimated 10,000 individuals distributed from 36–49°S in Chile [5] and from 39–43°S in Argentina [6]. Given their evasive behavior, this species remains unstudied in its natural habitat. Furthermore, it has been suggested that the pudú is being affected by landscape fragmentation and forest loss, predation by domestic dogs, competition with exotic species, and poaching activities [4, 7].

Generally spanning 16–23 ha [8], the home range of this species is quite restricted. The pudú feeds on several species of native shrubs and trees, eating the most nutritious parts including young leaves, buds, fruits, and flowers [7, 9]. As the only specialist deer of the temperate rainforest, this species likely plays a key role as a seed disperser [10, 11]. The restricted home range and its role as a seed disperser have led some authors to suggest that it is possible that viable pudú populations could be maintained within natural reserves [12, 13]; thus, some believe that the pudú is a viable target for conservation efforts.

Nationally managed protected areas have been an invaluable tool for in situ conservation [14]. These systems of protected areas have proven their effectiveness at protecting ecosystems and species with respect to the significant pressures of land-use change and land clearing [15]. The Chilean National System of Protected Areas (SNASPE in Spanish) has been a key instrument in determining wildlife conservation strategies in the country. Currently, the SNASPE is comprised of 100 management units distributed among 36 national parks, 49 national reserves, and 15 natural monuments. These units cover an area of 14.5 million hectares corresponding to 19.2 % of mainland Chile; thus, Chile’s current system of protected areas is above the 10 % protected ecosystems per country target threshold set by the Convention on Biological Diversity of the United Nations [16].

However, this system appears to be insufficient for the conservation of the fauna associated with temperate forests [17]. Since over 90 % of protected areas in Chile are concentrated at high latitudes (>43°), the areas with the highest species richness (35.6–41.3° S) remain largely outside the system. This, in turn, increases the risk of extinction not only due to the lack of protected areas in temperate forests but also because these areas are isolated within a mosaic of tree plantations, agricultural landscapes, and urban areas [17]. Although the pudú is commonly cited as the most common herbivore in national parks [18], there are no systematic records of its presence in these areas [7].

The conservation of a rare species is a difficult task considering the lack of information about current and potential habitats or habitat requirements [19]. Subsequently, predictive models, such as niche-based modeling, can be useful for obtaining reliable distribution maps to assess the suitability of proposed sites for conservation [20]. In this regard, the emergence of new mathematical methods for estimating potential distributions has complemented the lack of data [2, 21–23]. These methods often use the environmental characteristics of areas where a species is known to inhabit in order to estimate the environmental suitability of regions that currently lack records [24].

Currently, mathematical modeling of species distributions has several applications in conservation science including the prediction of geographic ranges of threatened or rare species [25–27], the identification of priority areas for conservation efforts [28, 29], the evaluation of extinction risk and/or suitable sites for reintroduction programs, and the implementation of wildlife corridors [20]. Considering the ecological importance of the pudú and the lack of up to date information concerning the distribution and conservation status of this species, in this study we estimate the distribution of the pudú within the National System of Protected Areas of Chile. Additionally, we evaluate the effectiveness of the system for the conservation of this species, define the priorities for new areas to protect, and evaluate the reduction of potential pudú habitat due to landscape fragmentation.

## Methods

### Study area

Although originally encompassing an area of 300,000 km^2^ [30, 31], the geographical range of the temperate rainforest of Chile and Argentina has been considerably reduced at geological time scales as a result of the advance and retreat of mountain glaciers during the Pleistocene [32], and in recent history due to large-scale human impacts on the landscape [33]. Now remnant vegetation covers just 30 % of the original area [31]. The current distribution of the temperate rainforest ranges from 38–49°S on the Chilean side of the Andes and adjacent areas of the provinces of Neuquén, Río Negro, and Chubut in Argentina [34]. The temperate rainforest has a Mediterranean pluviseasonal bioclimate and a temperate and sub-Mediterranean hyper-oceanic bioclimate [35].

The temperate rainforest of Chile encompasses the Valdivian Rainforest Ecoregion, which has been listed among the most endangered ecoregions of the world and has a critical conservation status [36, 37]. The high number of endemic birds (2 %), mammals (16 %), reptiles (62 %), amphibians (54 %) and plant species (49 %) makes this region biologically valuable. Hence, the Valdivian Rainforest Ecosystem is considered a hotspot of biodiversity and, therefore, a region of high conservation priority [31, 38, 39].

### Occurrence data

**Fig. 1:**
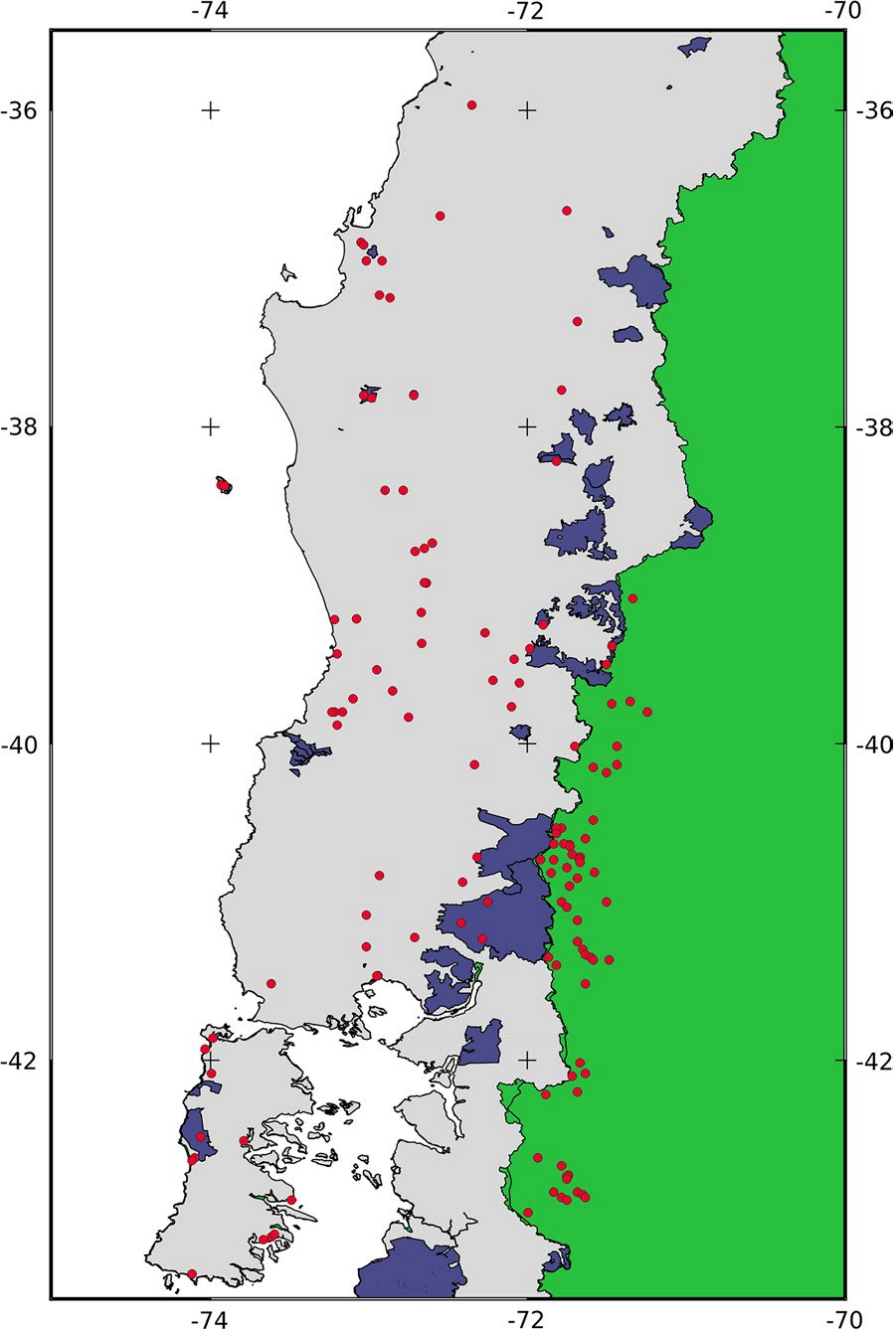
Georeferenced occurrence points of *P. puda* used for model fitting (*red*) in Chile (*light grey*) and Argentina (*green*). *Dark blue* polygons represent protected areas of Chile within the study area

Records of occurrence of the pudú were collected from several sources found in the literature [6, 8, 40, 41], as well as from databases of museum collections like MaNIS [42]. Points were considered only within the distribution range normally cited for Chile and Argentina [4, 5], since records of the southern area are controversial and their validity is unclear [41, 43]. In total, we considered 135 points of occurrence, of which 73 are located in Chile and 62 are located in Argentina (Fig. 1). In the event that the occurrences were not georeferenced (only location names), a standard procedure was followed whereby coordinates were assigned using the Gazetter GeoNames (http://www.geonames.org). Details of the presence points including source, year, and coordinates can be found in the Additional file 1.

### Environmental and geographic data

Global layers of the current weather conditions were obtained from the WorldClim database; the data have a spatial resolution of 2.5 arc—minutes (~5 km; [44]). These layers contain grouped variables collected monthly from 1950 to 2000. The variables used were selected according to previous modeling studies of other species of deer (e.g. *Odocoileus hemionus*; [45]). The following variables were used: average annual temperature (Ann T°), mean diurnal temperature range (MDR), temperature seasonality (T° S), maximum temperature of the warmest month (T° Max), minimum temperature of the coldest month (T° Min), annual precipitation (Ann Pp), seasonal precipitation (Pp S), precipitation over the wettest quarter (Pp Wet), precipitation of the driest quarter (Pp Dri), precipitation of the warmest quarter (Pp War), and precipitation of the coldest quarter (Pp Col). In addition to these variables, altitude (Alt) [44] was also incorporated in the analysis. The layers of the pudú distribution and the Chilean system of protected are were obtained from the IUCN [4] and the Chilean Forest Service (CONAF), respectively. Considering records of individuals moving up to 20 km in Argentina [46], and the fact that the pudú has an evolutionary history in the region, the study area used to train the model was defined by bounding the observed presences with a buffer of 100 km as a reasonable proxy of the area that has been accessible and probably explored [47, 48] by this small cervid. Processing of the environmental layers was performed in QGIS 2.10 and GRASS7.

### Statistical methods

A practical way to estimate the geographic distribution of a species is by characterizing the environmental conditions that are currently suitable for its persistence [49] and then identifying those areas where such conditions may be found [50]. A group of quantitative modeling approaches, known collectively as species distribution models (SDM), have been widely used to predict the potential geographic distribution of several animal species [24, 25, 49, 50]. Species distribution models are numerical tools that combine observations of species (either presence or presence and absence data) in a set of locations with environmental variables to obtain ecological and evolutionary insights and to predict distributions across landscapes [11, 15].

Considering that only presence data could be gathered to estimate the pudú geographical distribution, a maximum entropy approach was implemented in the Maxent 3.3.3 k software [51–53] as our ecological niche modeling approach. The Maxent model is a probability distribution selected by maximizing the entropy subject, which is constrained in that the expected value of each environmental variable under this uniform distribution should match the empirical value [51, 52]. The logistic model output represents the degrees of “habitat suitability”, ranging from 0 (not suitable) to 1 (suitable) [49]. The Maxent model was fitted using the default settings, and then it was evaluated using the AUC of the ROC curve and the “regularized training gain”. The ROC curve corresponds to the graph between 1—specificity (false positive rate) versus sensitivity (rate of true positives, [52]). The AUC measures the ability (probability) of the Maxent model to discriminate between presence sites and background sites [51, 54, 55]. The relative importance of each variable was estimated using the jack-knife method. First, the decrease in gain is calculated by adjusting the model using all variables except the focal variable and comparing this value with the gain of the full model (including all variables). Then, the model is fit using only the focal variable and comparing the gain with respect to the full model. The Maxent model results correspond to the average value of 20 replicas using a cross-validation framework [54, 56]. We used 20 replicates instead of the standard 10 just to increase our evaluation of the model’s predictive ability. The cross-validation scheme divides the dataset into 20 subsets. In each step, the model is fitted using 19 subsets and using the last dataset (independent) to test (validate) the fit. This procedure is repeated 20 times, and the AUC and jackknife values reported correspond to the average value of the 20 testing procedures.

### Post-processing

Our results are focused and restricted to Chilean habitats and protected areas. The fitted model, trained in the study area, was later projected to Chile, to estimate the distribution of the species. The original map was converted to a binary map (0 = no-suitable, 1 = suitable), applying a threshold that maximizes sensitivity and specificity [57] to obtain a balance between commission and omission errors. Then, the percentage of the area contained in the public protected areas was calculated for the distribution map (Fig. 1). In addition, the percentage of area containing the pudú distribution proposed by the IUCN was calculated.

## Results

**Fig. 2:**
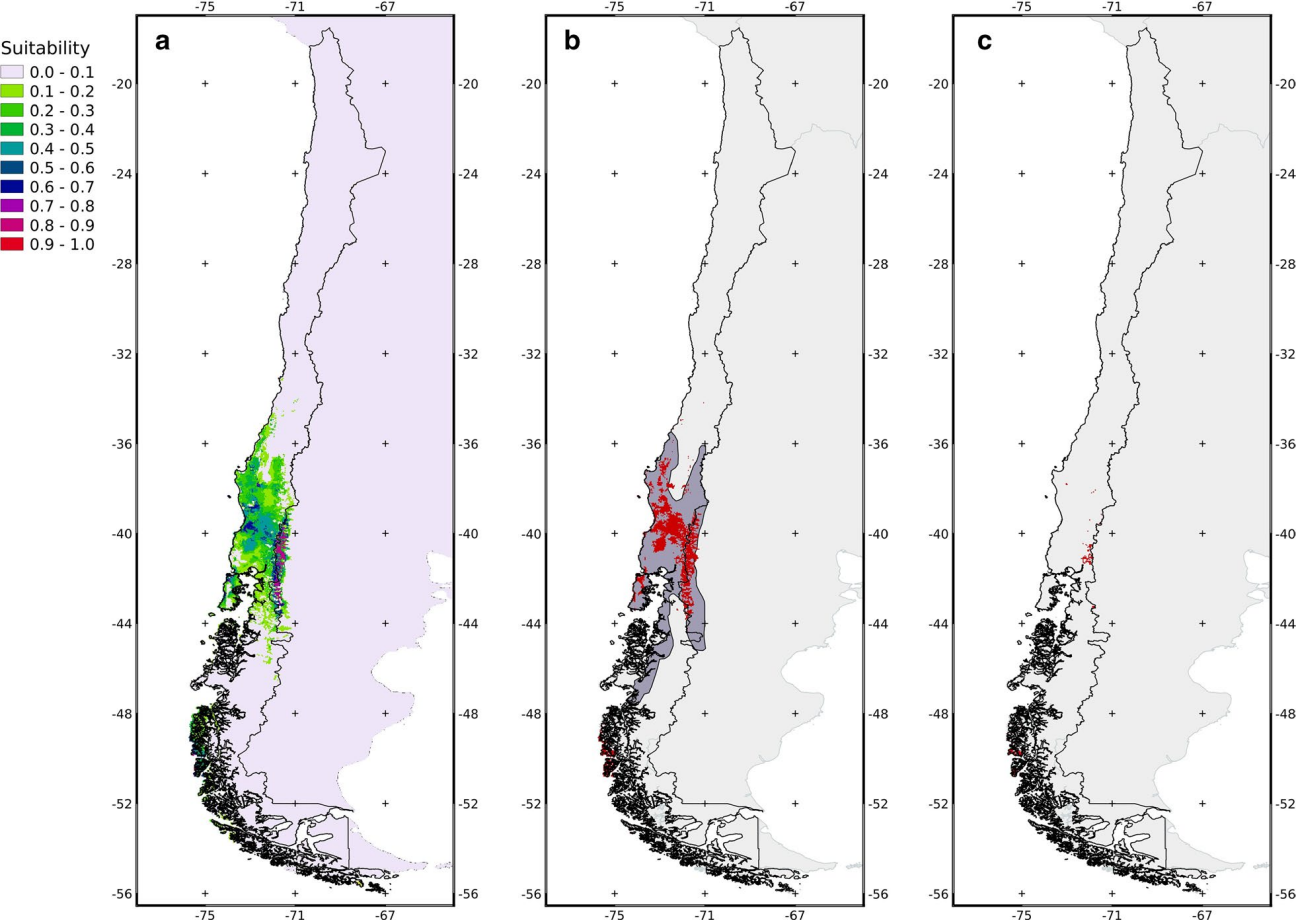
Model results: **a** Projection of the model fitted for the Chilean territory. *Colors* represent the suitability of each pixel for *P. puda* habitat. **b** Binary map of the projection of the model fitted for the Chilean territory (*red*) with respect to the distribution determined by the IUCN (*dark grey*) for *P. puda.* **c** Overlap areas between *P. puda* suitable areas and protected areas according to the model (*red*)

The most suitable areas for the pudú were restricted to the central valley (between the Andes and the Cordillera de la Costa) at low altitudes in the Andes from 38.67 to 39.81°S and in some isolated patches from 42.02°S southwards (Fig. 2a). The AUC for the model was 0.818, indicating a high predictive capacity [55]. The most important variables, according to their effect on the training gain (decrease), were seasonal temperature and mean diurnal temperature range (Table 1). Furthermore, the analysis of the models including only one variable showed that the models fitted with only seasonal temperature, precipitation of the driest quarter, and mean diurnal temperature range, had the highest values of gain (Table 1). The threshold that maximizes sensitivity and specificity was 0.3591, which was used to obtain the binary maps.

**Table 1:** Jackknife statistics of model performance and relative importance of each variable

Environmental variables	Training gain
Alt	0.942†−0.120
Ann T°	0.938†−0.101
Ann Pp	0.955−0.295
Pp S	0.946−0.240
Pp Wet	0.952−0.254
Pp Dri	0.955−0.325‡
Pp War	0.956−0.326‡
Pp Col	0.949−0.257
MDR	0.903†−0.328‡
T° S	0.879†−0.326‡
T° Max	0.948−0.118
T° Min	0.951−0.068

For each variable, the first value corresponds to the gain of a model fitted using all variables except the focal one. The most important variables according to this criterion are marked with †

The second value corresponds to the gain of a model fitted using only the focal variable. The most important variables according to this criterion are marked with ‡ (see “Methods” for details)

The pudú distribution predicted by the Maxent model had an estimated area of 79,047 km^2^ (33,934 km^2^ in Chile, 43 %), 37,722 km^2^ (29.4 %) of which matched the IUCN estimated distribution of 128,278 km^2^ (Fig. 2b). The area of the model-predicted distribution contained in Chilean protected areas is almost entirely located at high altitudes of the Andes. There was only 4644 km^2^ of overlap between the predicted distribution of the pudú and area currently being protected in Chile; this overlap represents only 5.87 % of the complete distribution of the pudú (Fig. 2c).

## Discussion

The estimated distribution of the pudú calculated here is almost entirely contained within the area described by the IUCN for this species [4], however the predicted distribution is less extensive in size. The predicted distribution lies within the Valdivian Rainforest Ecoregion, which is consistent with the habitat preferences previously described for this temperate rainforest associated species [5, 6, 39]. Studies have noted that this species prefers rainforest habitats where it can find shelter and highly nutritional food [7, 9]. The most suitable habitats for the pudú were located in the central valley (central depression) of Chile. This area is currently a highly fragmented landscape with isolated populations that are vulnerable to strong anthropogenic pressures [58]. Furthermore, in this area the system of public protected areas (SNASPE) has low coverage making the viability of these remnant populations more difficult (Fig. 2c). Landscape fragmentation in the central valley has resulted in the degradation of the most favorable pudú habitats. As has been found for other species, this fragmentation has forced relict populations to persist in the periphery of their historical geographic range [59].

The model supports the presence of the pudú in the 14 protected areas cited in the literature: the Vicente Perez Rosales, Puyehue, Villarrica, Tolhuaca, Conguillo, and Chiloé National Parks; and the Nonguen, Ñuble, Altos de Pemehue, Isla Mocha, Huerquehue, Mocho Choshuenco, Alerce Costero, and Futaleufu National Reserves (Figs. 1, 2c). The percentage of the predicted pudú distribution in protected areas was low, and in most cases these were not considered to be the most suitable areas for the species according to our models. Due to the fact that the majority of the overlay between pudú favorable habitat and protected area being located in the Andes, together with the low representation of protected areas in the central valley and the coast, imply a major threat to the species as a result of isolation between these populations [60–62]. Suitable areas outside the protected parks represent an opportunity for the conservation of this cervid. Approximately 14,000 km^2^ of the area around the parks houses broadleaved forests in different stages of conservation [63] that are currently being used for pudú corridors and refuges. However, the high fragmentation of these lands makes coordinating conservation efforts difficult.

The most important variables affecting the estimated distribution of the pudú were the mean diurnal temperature range and temperature seasonality (these variables were selected using two different criteria). According to the models, mean diurnal temperate range was negatively related with habitat suitability. This suggests that the pudú is intolerant to sudden changes in temperature throughout the day and is probably better adapted to tolerate low rather than high temperatures. This is in agreement with previous studies, which indicate that the pudú is less active when the sun’s intensity is highest and instead is more active during sunrise and sunset [8]. The same negative effect of sunlight or temperature has been described for the kudu (*Tragelaphus strepsiceros)*, elk (*Cervus canadensis*), and other deer species (i.e. *Odocoileus* spp.) [64]. Moreover, some authors suggest that ungulate species inhabiting temperate climates would have a lower tolerance to high temperatures than ungulates inhabiting non-temperate climates [64, 65]. On the other hand, the relationship between temperature seasonality and habitat suitability found in this study was irregular, but in general it has been suggested that the pudú can tolerate moderate seasonal variation in temperature, which could be due to the relationship between temperature and the availability of vegetation that it feeds on [66, 67].

The current condition of the pudú within the Chilean system of protected areas remains unknown; however, it has been suggested that even within national parks and reserves, there are still anthropogenic threats such as the presence of feral dogs and domestic livestock [68]. The categorization of the species as Vulnerable according to the IUCN seems to be appropriate given the low representation of suitable habitat within protected areas and the limited amount of information on the status of wild populations. It is therefore crucial to implement new protected areas within the central valley, which could serve as corridors to reduce the rate of species extinctions and increase the likelihood of the re-colonization of parks [60, 67]. The importance of the pudú extends beyond the species’ ecological role. As the pudú is a small charismatic species, this deer is a good candidate flagship species that could help attract public attention and sympathy to the conservation of these important habitats, and this, in turn, would likely be of benefit to other species [68].

## Conclusions

Overall, the results of this study indicate that the habitat of this endangered cervid is poorly represented in the Chilean system of nationally protected areas. Habitat within this system only represents marginal (less suitable) sites of the pudú’s original distribution. Currently, the more suitable areas, in the central valley of Chile, are highly fragmented and used for agricultural, forestry, or other human activities. This highly fragmented land may be a major obstacle for conservation efforts. Initiatives such as new protected areas (public and private), feral dog control, habitat conservation, among others, will contributed to the viability of the small remnant populations of pudú in the central valley.

## Additional file

10.1186/s12898-015-0055-7 List of the presence points used in this study. The table includes information source, year recorded, and coordinates.

## Abbreviations

IUCN: International Union for Conservation of Nature
SNASPE: Chilean National System of Protected Areas
MaNIS: The Mammal Networked Information System
AUC: area under the curve
ROC: receiver operating characteristic
